# Retinoic acid enhances germ cell differentiation of mouse skin-derived stem cells

**DOI:** 10.1186/s13048-018-0390-3

**Published:** 2018-03-01

**Authors:** Paul W. Dyce, Neil Tenn, Gerald M. Kidder

**Affiliations:** 1 Department of Animal Sciences, College of Agriculture, Auburn University, CASIC Building, 559 Devall Drive, Auburn, AL 36849 USA; 2 Department of Physiology and Pharmacology, The University of Western Ontario and Children’s Health Research Institute, 800 Commissioners Road East, London, ON N6C 2V5 Canada

## Abstract

**Background:**

Retinoic acid (RA) signaling has been identified as a key driver in male and female gamete development. The presence of RA is a critical step in the initiation of meiosis and is required for the production of competent oocytes from primordial germ cells. Meiosis has been identified as a difficult biological process to recapitulate in vitro*,* when differentiating stem cells to germ cells. We have previously shown that primordial germ cell-like cells, and more advanced oocyte-like cells (OLCs), can be formed by differentiating mouse skin-derived stem cells. However, the OLCs remain unable to function due to what appears to be failure of meiotic initiation. The aim of this study was to determine the effect of RA treatment, during stem cell differentiation to germ cells, particularly on the initiation of meiosis.

**Results:**

Using qPCR we found significant increases in the meiosis markers *Stra8* and *Sycp3* and a significant reduction in the meiosis inhibitor Nanos2, in the differentiating populations. Furthermore, OLCs from the RA treated group, expressed significantly more of the meiosis regulatory gene *Marf1* and the oocyte marker *Oct4*. At the protein level RA treatment was found to increase the expression of the gap junction protein CX43 and the pluripotency marker OCT4. Moreover, the expression of SYCP3 was significantly upregulated and the localization pattern better matched that of control fetal ovarian cells. RA treatment also improved the structural integrity of the OLCs produced by initiating the expression of all three zona pellucida transcripts (*Zp1–3*) and improving ZP3 expression levels and localization. Finally, the addition of RA during differentiation led to an almost two-fold increase in the number of OLCs recovered and increased their in vitro growth.

**Conclusion:**

RA is a key driver in the formation of functioning gametes and its addition during stem cell to germ cell differentiation improves OLCs entry into meiosis.

## Background

During early embryogenesis mouse primordial germ cells have the potential to either develop to form spermatogonia or begin the process of meiosis and develop into oocytes. In mammals, meiosis onset begins before birth in females, or at the onset of puberty in males. The first evidence of entry into meiosis I can be seen at ~ 13.5 days postcoitum (dpc) in the female fetal mouse [1]. This decision has been shown to be influenced by the presence of retinoic acid (RA) which is produced by mesonephroi during embryogenesis in both sexes [2]. Production of the premeiotic gene *stimulated by RA 8* (*Stra8*) precedes meiosis initiation at ~ 12.5 dpc [2–4]. Conversely, in the male the activity of RA is stopped through the action of the retinoid-degrading enzyme cytochrome P450 26B1 (CYP26B1) [5]. Testicular germ cells do not enter meiosis during fetal development and *Stra8* expression is first identified 10 days postpartum, concurrent with the onset of meiosis [6]. In recent years, independent investigations have resulted in RA emerging as a key driver for the entry of both male and female germ cells into meiosis [2, 5, 7–10].

Previous studies have shown that media containing growth factors, including RA, are able to sustain mouse germ cells in the absence of somatic cells and allow them to enter into and progress through meiotic prophase I, in the absence of leukemia inhibitory factor (LIF) [2, 11, 12]. Three initial publications demonstrated the induced differentiation of ES cells into oocytes or sperm, though failed to show functioning gametes [13–15]. We have also shown that skin-derived somatic stem cells, from pigs, mice and humans, have the ability to form primordial germ cell-like cells (PGCLCs) and non-functioning oocyte-like cells (OLCs) [16–21]. The OLCs were characterized by their morphology and expression of oocyte markers but have yet to fertilize correctly and function. The failure of OLCs, produced from somatic stem cells, appears to involve a failure to properly initiate and complete meiosis. Recent studies, differentiating ES cells, have included an RA induction phase and resulted in completion of meiosis [22, 23]. ES cells originate from the inner cell mass of developing blastocysts. Therefore, ES cells used for cell therapy are allogenic with the transplanted donor cells not originating from the recipient. This raises the concern of immunogenic response from the host. Moreover, the use of ES cells is impeded by moral, legal, and ethical concerns.

The increased utility provided by the use of somatic stem cells illustrates the necessity for continued investigation of their differentiation capabilities. We hypothesize that the addition of RA during induced differentiation will enhance the ability of skin derived stem cells to develop into OLCs. Therefore, in this study we investigated the use of RA to improve the generation of OLCs from mouse skin-derived somatic stem cells and its ability to improve the induction and progression of meiosis in the OLCs produced.

## Methods

### Stem cell isolation and culture

All experiments involving animals in the study were conducted according to the Care and Use of Experimental Animals Guidelines of the Canadian Council on Animal Care, and have been approved by the Western University Animal Care and Use Committee. Newborn female transgenic mice [Jackson Lab; 004654; (CBA/CaJ X C57BL/6 J)F2] carrying the *Oct4- GFP* transgene were euthanized within 24 h of birth and the dorsal skin removed. Skin stem cells were isolated using a protocol by Toma et al. with the following modifications [24]; Skin samples from 4 to 5 pups were grouped and placed in Hank’s balanced salt solution (HBSS, Thermo Fisher Scientific) and cut into ~ 1 mm square pieces using dissecting scissors. The samples were then washed 3X using HBSS, and re-suspended in 1 ml of 0.05% trypsin for 40 min. at 37 °C. Following trypsinization, 1 ml of 0.1% DNase (Sigma) was added to the sample and incubated 1 min. at room temperature. Then 9 ml of HBSS was immediately added and the cells pelleted at 500 X G for 5 min. Samples were then washed 1X with HBSS and 2X with DMEM-F12 with antibiotics (Thermo Fisher Scientific). Following the last wash, the samples were mechanically dissociated in 1 ml of DMEM-F12 by pipeting. The partially dissociated samples were then filtered using a 40 μm cell strainer (BD Falcon). This was done by adding 9 ml DMEM-F12 to the dissociated cells and running them through the filter. This was followed by 10–15 ml of DMEM-F12. The resulting filtrate was then pelleted by centrifuging for 5 min. at 500 X G. Each pellet obtained from 4 to 5 pups was then re-suspended in 10 ml stem cell medium (DMEM-F12 with 1 X B27 (Thermo Fisher Scientific), 20 ng/ml epidermal growth factor (EGF, Sigma), and 40 ng/ml basic fibroblast growth factor (Sigma)) and plated on a 10 cm dish (Sarstedt). At ~ 72 h after plating, the skin-derived stem cells grew as suspended spheres, which discriminated them from the rest of the skin cells (attached) in culture. To passage floating cell spheres, medium containing spheres was centrifuged and the pellet was gently dissociated using a large bore pipette. The cells were re-seeded in fresh stem cell medium as above. Cells were passaged every 4–6 days.

### Stem cell differentiation

The isolated stem cells at passage two were pelleted at 500 X G and re-suspended in 500 μl of phosphate-buffered saline (PBS). The cells were dissociated to single cells by using vigorous pipetting. The cells were then washed in 9 ml of PBS and counted on a hemocytometer. For the stem cell only group (SC), and ovarian cell only groups, cells were plated at 0.6 X 10^6^ cells per well (500 μl) in differentiation medium which consists of TCM199 (no antibiotics, Thermo Fisher Scientific) supplemented with 0.05 IU follicle stimulating hormone (Sioux Biochemical), 0.03 IU luteinizing hormone (Sioux Biochemical), 3 mg/ml bovine serum albumin (BSA, Sigma), 5 μl/ml ITS (Life Technologies), 0.23 mM sodium pyruvate (Life Technologies), 1 mg/ml Fetuin (Sigma), and 1 ng/ml EGF (Sigma). Cells were cultured at 37 °C for 12 days, changing half the medium every 2 days. Spent medium was centrifuged and the pelleted cells returned to the culture dish. A 10 mM RA stock solution was prepared by diluting the RA in dimethyl sulfoxide (DMSO, Sigma). During the differentiations, at the beginning of day 4, cultures were treated with either 10 μm RA or treated with the same concentration of dimethyl sulfoxide (DMSO, vehicle only control) for 24 h. At the end of the 12 days of differentiation, the aggregates were trypsinized and large cells collected using a stereoscope and mouth pipette.

### Western blot

For immunoblotting, protein from differentiated cells and adult ovary samples were isolated using radio-immunoprecipitation assay (RIPA) lysis buffer with complete mini protease inhibitors (Roche) added fresh prior to use. 30 μg of protein (as determined using a BSA protein assay kit, Pierce) were mixed with 5X reducing sample buffer, boiled for 5 min, and electrophoresed under reducing conditions on 12% polyacrylamide gels. Protein was transferred using an iBlot (Thermo Fisher Scientific) onto nitrocellulose membranes. Membranes were incubated for 2 h in 5% non-fat dry milk blocking buffer at room temperature, followed by an overnight incubation at 4 °C in primary antibody (HSP70 1:5000; Chemicon, CX43 1:10000; Sigma, OCT4 1:300; Santa Cruz Biotech). After a 1 h incubation with anti-mouse IgG (alexa 680; 1:10000; Thermo Fisher Scientific) or goat anti-rabbit (Alexa 488; 1/1000; Thermo Fisher Scientific) at room temperature, expression was detected following three washes, using the LI-COR Odyssey fluorescent scanner. Blots were incubated in 1X strip buffer (re-blot plus mild, Millipore) at room temperature for 15 min and re-blocked 1 h between antibody staining. All blots were stripped, blocked, and re-probed between antibodies.

### RNA isolation and RT-PCR

RNA was isolated using the RNeasy Mini Kit (Qiagen) according to the manufacturer’s protocol. RT-PCR on differentiated cultures was performed as previously described [20]. Briefly, RT-PCR on groups of 15 OLCs or oocytes was performed by freezing cells in 7 μl of lysis buffer containing 14 U of porcine RNase inhibitor (Amersham) and 5 mM DTT (Thermo Fisher Scientific) at –80 °C until use. Cells were then lysed by boiling for 1 min and vortexing for 2 min (repeated for a total of 3X), and then stored on ice. Samples were then DNase treated by adding 1 μl 10X DNase buffer and 1 μl amplification grade DNase (Thermo Fisher Scientific) and incubating 15 min. at room temperature. 1 μl EDTA (25 mM) was then added and the samples were incubated for 10 min. at 65 °C. RT was then performed by adding 0.5 μl H_2_O, 5 μl 5X buffer, 1.25 μl of random hexamer primers, 6.25 μl 2 mM dNTPs, and 1 μl MMLV reverse transcriptase to the sample. The samples were incubated at 25 °C for 10 min., 37 °C for 50 min., and 70 °C for 15 min. Real-Time PCR was carried out on a Smart Cycler (Cepheid) by using the QuantiTect SYBR green PCR kit (Qiagen): 2.5 μl, for cell populations, and 3.1 μl for groups of 15 cells, of DNase treated cDNA (from a 25 μl RT reaction) was added to 12.5 μl of SYBR green mix and 0.3 μM each of forward and reverse primers (final volume 25 μl). The housekeeping gene hypoxanthine guanine phosphoribosyl transferase (Hprt) was used to calculate the relative transcript levels of target genes using the ΔΔCT method. Product sizes were confirmed on a 1% agarose gel. The identities of the products were confirmed by sequencing.

### Immunofluorescence

Cells were washed twice with PBS and fixed in 4% paraformaldehyde in PBS for 20 min. Cells were then washed three times in PBS with 0.1% Tween 20 and incubated for 10 min, and then for 20 min in PBS with 1% Triton-X-100. Next, cells were blocked for 2 h in PBS with 5% BSA, and 0.05% Triton-X-100 (PBS-B, blocking solution), followed by an incubation with primary antibody, 1:400 anti-ZP3 (Sigma) or 1:300 anti OCT4 (Santa Cruz Biotech) for 2 h at 37 °C, or overnight at 4 °C. Cells were then washed in blocking solution (PBS with 5% BSA and 0.05% Triton X 100), and incubated with 1:1000 PE-conjugated goat anti-rabbit IgG or 1:500 FITC-conjugated rat anti-mouse IgG for 1 h at room temperature. This was followed with a blocking solution wash and incubation with 4′-6-diamidino-2-phenylindole (DAPI) for 1 min, followed by washing three times with PBS-B. Cells were mounted using fluorescent mount medium (DakoCytomation) and viewed using an Olympus BX-UCB microscope and MetaMorph analysis software (Universal Imaging Corporation).

### Chromosome spreading and SYCP3 staining

At day 6 cells from the RA treated or control differentiations were trypsinized (Thermo Fisher Scientific) for 3 min at 37 °C, washed once in DMEM H21 (Life Technologies) with 10% fetal calf serum (FCS, Life Technologies) and then resuspended in 0.5 ml PBS. Thirty μl of the suspension was then placed on a slide and incubated for 5 min at room temperature to attach the cells to the slide surface. To spread the cells, 90 μl of 3% sucrose was gently added to the slide which was then held for 20 to 30 min at room temperature. Fixative was prepared as 2% paraformaldehyde with 2 μl of 10% sodium dodecyl sulfate (SDS, Life Technologies) per ml. The pH was adjusted to between 9 and 11 by adding 10 μl NaOH (1 N) per 8 ml of fixative followed by 3 rinses in PBS block with tween (PBT), each for 10 min at room temperature. PBT was made by adding 0.15 g BSA and 100 μl Tween20 to 100 ml PBS. The primary antibody against synaptonemal complex protein 3 (SYCP3, 1/100) was then added and the slides were incubated in humidity chambers overnight at 4 °C. Prior to adding anti-rabbit FITC (1/500; Abcam), the slides were washed three times with PBT for 10 min in each wash. The slides were then incubated for 2 h at room temperature, then washed twice with PBT for 10 min at room temperature. Slides were stained with Hoechst (Life Technologies) for 7 min, washed once in PBT, and then mounted using fluorescent mounting medium (Dako, S3023). Slides were viewed using an Olympus BX-UCB microscope and MetaMorph analysis software (Universal Imaging Corporation).

### Statistical analysis

Experiments were repeated at least three times and the data were analyzed using a t-test (on comparison of germ cell marker expression levels between OLC and the oocyte groups) or ANOVA, followed by the Tukey test. Results were considered significant at *P* < 0.05.

## Results

### Treatment of differentiations with RA results in altered expression of meiosis markers

**Fig. 1:**
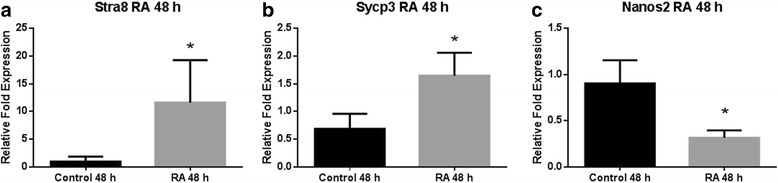
RT real-time PCR analysis of meiosis related gene expression in differentiation cultures exposed to RA or vehicle only for 48 h. **a** The expression of *Stra8* was significantly higher (11.63 ± 7.64 fold) following exposure to 10 μm RA when compared to the vehicle only control. **b** Similarly, the expression of *Sycp3* was significantly higher (1.65 ± 0.42 fold) following exposure to 10 μm RA when compared to the vehicle only control. Conversely, the expression of the meiosis inhibitor gene *Nanos2* was significantly lower, 0.32 ± 0.08 fold when compared to the vehicle only contol. * denotes significant difference *p* < 0.05 using a t-test.

Initially, in order to determine the induction of meiosis we compared transcript levels of the meiosis marker genes *Stra8* and *Sycp3*. We found the expression of *Stra8* was significantly higher, at 11.63 ± 7.64 fold, following RA treatment (*p* < 0.01, Fig. 1a). Similarly, the expression of *Sycp3* was significantly increased 1.65 ± 0.42 fold higher than the vehicle only control (*p* < 0.05, Fig. 1b). Finally, we compared the expression of the gene encoding the meiosis inhibitor NANOS2 and found significantly less was present following the treatment with RA (*p* < 0.05). The RA treated groups expression decreased to 0.32 ± 0.08 fold the level of that in the vehicle only control, 0.90 ± 0.25 fold (*p* < 0.05, Fig. 1c).

### Treatment with RA results in increased expression of Marf1, Oct4, and Sycp3 genes in OLCs

**Fig. 2:**
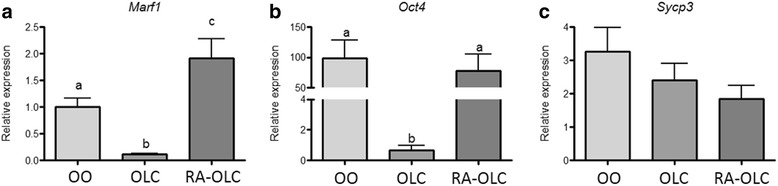
RT real-time PCR analysis of the relative gene expression of *Marf1* (**a**), *Oct4* (**b**), and *Sycp3* (**c**; a pluripotency marker) normalized to *Actb*. Groups of 15 OLCs or oocytes per sample were tested from at least 3 biological replicates. Treatment with RA increased the expression of *Marf1* in OLCs (**a**). No significant difference was observed between oocyte and OLC groups for *Sycp3* (**c**). There was a significantly higher level of *Oct4* mRNA when comparing oocytes and RA treated OLCs to untreated OLC groups (**b**). The data were analyzed using one-way ANOVA with Tukey’s post-hoc test (data represent at least *n* = 3 biological replicates)

We next set out to test the expression of oocyte specific markers in OLCs produced from RA treated and vehicle only controls. Groups of 10 OLCs produced either with or without RA treatment were compared using qRT-PCR. The expression of the gene encoding meiosis regulator and mRNA stability factor 1 (MARF1) was found to be lower than in natural oocytes in our control differentiations. The treatment with RA resulted in the expression of *Marf1* significantly increasing to higher levels than the untreated control and the natural oocyte control (*p* < 0.05, Fig. 2a). Conversely, the expression of the pluripotent marker gene *Oct4,* in our control OLCs, was found to be significantly lower than in our oocyte controls (*p* < 0.05, Fig. 2b). OLCs produced following RA treatment expressed significantly higher *Oct4* mRNA, not significantly different from that of control oocytes (*p* > 0.05, Fig. 2b). Finally, we tested the expression of the meiosis marker gene *Sycp3*. We did not see a significant difference in the expression of *Sycp3* in either our treated or untreated OLCs nor in the control oocytes (*p* > 0.05, Fig. 2c).

### Treatment with RA results in increased CX43 and OCT4 expression

**Fig. 3:**
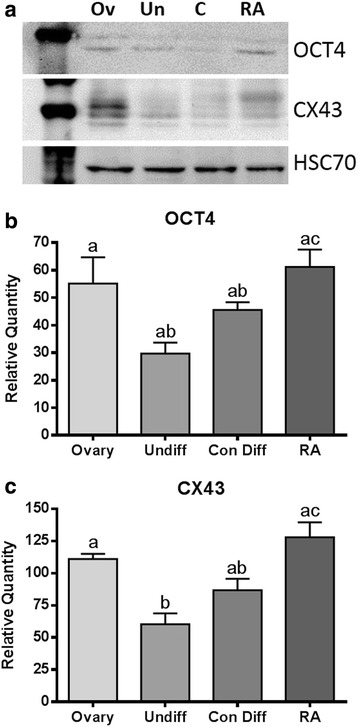
Western blot analysis of the protein expression levels in differentiation cultures of the pluripotency marker OCT4 and the gap junction protein CX43. **a** Representative blots depicting the expression levels of OCT4 and CX43 with HSC70 used as a loading control in ovary (Ov), undifferentiated stem cells (Un), untreated control differentiations (**c**), and RA treated differentiations (RA). **b** While there was no significant difference in the expression of OCT4 between the control (Con Diff) and RA exposed differentiations (RA), the expression of OCT4 in the RA treated group was found to be significantly higher than in the undifferentiated control group (Undiff). **c** The expression of CX43 was found to be significantly higher in the RA treated differentiation when compared to the vehicle only control differentiations. In both cases the expression level of CX43 was found to be not significantly different from adult mouse ovary (Ov) controls. The data were analyzed using one-way ANOVA with Tukey’s post-hoc test (Data represent at least *n* = 3 biological replicates)

The requirement of cellular cooperation between granulosa and the oocyte, through the presence of connexin 43 (CX43) based gap junctions, for the correct development of oocytes has been well defined (for review see [25]). Therefore, we were interested in investigating the effect of RA on the expression of CX43 and the oocyte marker octamer-binding transcription factor 4 (OCT4). We compared the expression of OCT4 and CX43 at the protein level using Western blotting. The expression of OCT4 was significantly higher in the RA treated differentiations when compared to the vehicle only control (*p* < 0.05, Fig. 3a and b). Similarly, the expression of the gap junction protein CX43 was found to be significantly higher following treatment with RA when compared to the control untreated differentiations (*p* < 0.01, Fig. 3a and b).

### The localization of SYCP3 is improved following treatment with RA

**Fig. 4:**
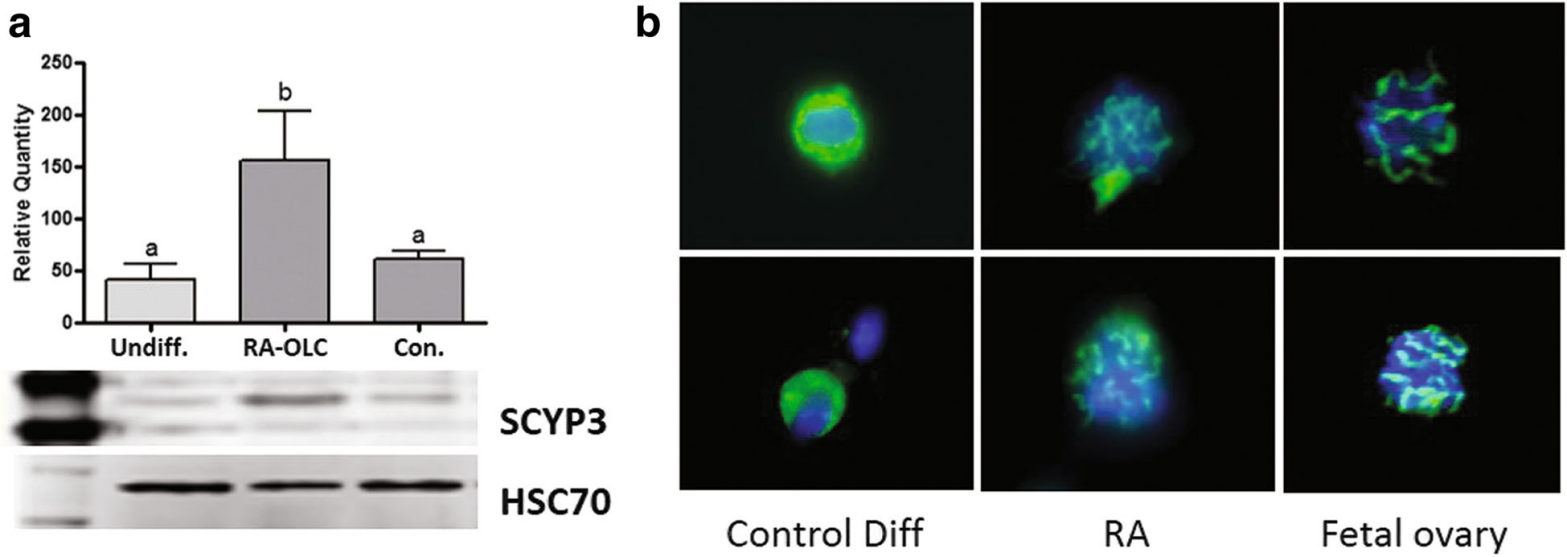
Analysis of SYCP3 expression following treatment of differentiations with RA for 48 h. **a** The expression level of SYCP3 was found to be significantly higher following exposure to 10 μm RA when compared to the vehicle only control or undifferentiated stem cells. **b** Immunolocalization of SYCP3 had a largely cytoplasmic localization in control differentiations (left two panels). In the RA treated cultures the location of SYCP3 (middle two panels) more closely resembled that of fetal ovary controls (right two panels), with chromosome localization. The data were analyzed using one-way ANOVA with Tukey’s post-hoc test (Data represent at least *n* = 3 biological replicates)

In order to determine if the PGCLCs were entering meiosis we utilized Western blotting to detect the expression level of the synaptonemal complex protein SYCP3. The overall expression of SYCP3 was found to be significantly higher following treatment with RA when compared to the undifferentiated or differentiated cells exposed to vehicle only (*p* < 0.05, Fig. 4a). We next utilized immunofluorescent staining for SYCP3 in chromosomal spreads to determine the localization. The majority of cells stained in the control culture had cytoplasmic SYCP3 staining (Fig. 4b). This was contrasted dramatically by stained control female fetal gonad spreads which had the phenotypically characteristic synapsed staining pattern of SYCP3. Following treatment with RA the SYCP3 staining more resembled the positive control staining with SYCP3 at least partially synapsed with the DNA.

### RA treatment improved the formation of the zona pellucida

**Fig. 5:**
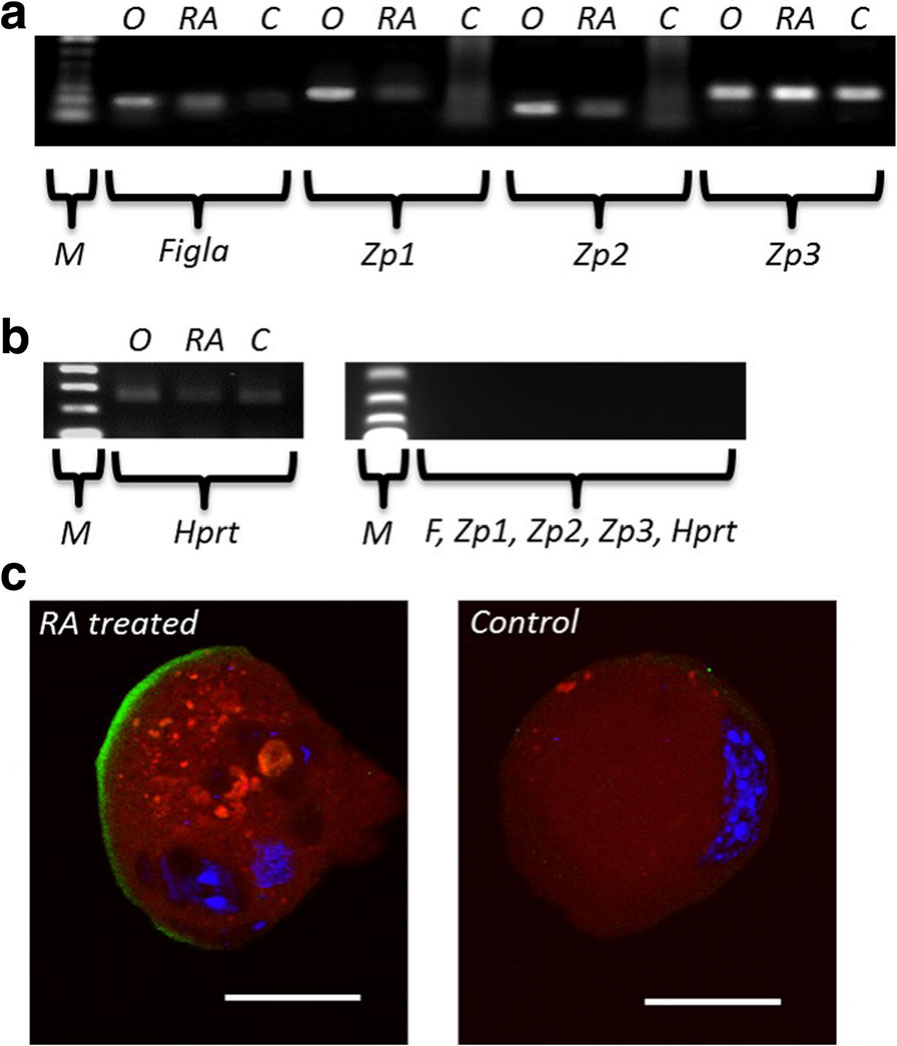
The expression of *Figla*, and *Zp1-Zp3* was tested in the differentiations using RT-PCR (**a**). The gel is loaded with 1 kb marker (M) followed by PCR products from ovary (O), RA treated (RA), and control (**c**) differentiations repeated for the *Figla* and *Zp1–3* transcripts (**a**). **b** Loading controls for the PCR reactions. Hprt was usied as a positive loading control (left panel). Primer only negative controls, loaded left to right 1 kb marker (M), Figla (F), Zp1–3, and Hprt, using water to replace cDNA (right panel). **c** Representative immunofluorescent images depicting the expression of OCT4 (red) and ZP3 (green) in RA treated (left panel) and control OLCs (right panel). **b** Scale bars = 30 μm

In our earlier work we found that the OLCs are much more fragile than natural oocytes [17–20]. We have previously shown that this may potentially be due to the failure of OLCs to produce ZP1 and ZP2, components of the zona pellucida [19]. We utilized RT-PCR to determine if the treatment of the differentiations with RA improved the expression of the genes *Figla*, *Zp1*, *Zp2*, and *Zp3*. We found that the treatment of the differentiations with RA resulted in the expression of all four transcripts while the control differentiation failed to express *Zp1* and *Zp2* (Fig. 5a). Moreover, while the protein expression of ZP3 was membrane localized in both OLCs from untreated and RA treated differentiations, the expression was more intense and continuous in the OLCs from RA treated cultures (Fig. 5c). It is important to note that the ZP membrane, while improved with the RA treatment, remains not as strong in the OLCs (unpublished results).

### RA treatment improves the efficiency of OLC generation from skin derived stem cells

**Fig. 6:**
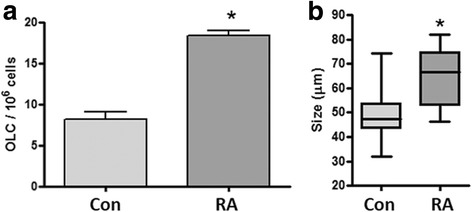
Analysis of OLC production and growth efficiency. **a** The number of OLCs was determined from starting cell numbers with or without RA treatment. There were significantly more OLCs produced in the RA treated cultures. **b** Over the 6 day differentiation, OLC in the RA treated (left panel)) group reached a larger size when compared to those recovered from the vehicle only control differentiation (right panel). * denotes significant difference *p* < 0.05 using a t-test.

We next compared the number of OLCs generated per 10^6^ starting cells. RA treatment was found to increase the number of OLCs from 8.17 ± 2.22 in the control differentiations to 18.39 ± 1.58 per 10^6^ cells (*p* < 0.01, Fig. 6a). Recovered oocytes were measured and found to be on average significantly larger in the RA treated group (49.25 ± 11.66 μm, *p* < 0.01) when compared to the untreated control OLCs (68.75 ± 5.94 μm, Fig. 6b).

## Discussion

Currently, many stem cell types have been demonstrated to have the potential to form germ cells in vitro (for review see [26]). However, there have been very few examples of the germ cells produced successfully initiating and completing meiosis, indicating a lack of competency for efficiently producing gametes from stem cells in vitro. The ability to produce functioning gametes from stem cells will provide a closed system in which to study germ cell formation and development.

Retinoic acid, the biologically active form of vitamin A, was initially identified as a requirement through nutritional studies (for review see [27]). Recent investigations have shown that RA is involved in meiosis initiation and is an important regulator of many organ systems during development (for review see [28]). We reasoned that for the successful production of a female gamete the presence of RA would be required to properly enter the meiotic process. Therefore, we initially treated our differentiations with RA while monitoring the expression level of genes critical for meiosis. We found the early meiosis marker *Stra8* and the later meiosis marker *Sycp3* were both significantly upregulated following the RA treatment. We next wanted to test the expression of the RNA-binding protein *Nanos2,* which has been shown to inhibit meiosis entry in the mouse by suppressing the expression of *Stra8* [29]. Interestingly, we found treatment of the differentiations with RA resulted in significantly decreased expression of *Nanos2* that coincided with the increased expression of *Stra8* (Fig. 1). This suggested that, as a whole population, the differentiations were responding to RA treatment by removing a meiosis inhibiting factor *Nanos2* and increasing the meiosis initiation factor *Stra8,* suggesting an increased population of meiosis competent cells. The increased expression of the meiosis marker *Sycp3* further supported this finding.

We next compared the expression of oocyte markers specifically in the OLCs. To do this we took groups of 15 RA treated and untreated OLCs and compared them to groups of 10 natural mouse oocytes. We initially tested the expression levels of *Marf1*, a recently identified marker important for controlling meiosis and the maintenance of genomic integrity [30, 31]. The mutation of *Marf1* results in large scale alterations to gene expression and a failure of meiosis in oocytes [30]. *Marf1* was found to be expressed at a significantly lower level in our untreated, control differentiation produced, OLCs when compared to natural oocytes (Fig. 2a). OLCs produced following RA treatment had significantly higher *Marf1* expression than untreated OLCs and natural OLCs (Fig. 2a). Further study will be required to determine if a higher expression of *Marf1* leads to issues with progression through meiosis. Similarly, *Oct4* expression was found to be significantly lower in the untreated OLCs when compared to natural oocytes (Fig. 2b). The RA treated OLCs had a similar expression level of *Oct4* compared to natural oocytes (Fig. 2b). Interestingly, the expression of *Sycp3* was not found to be significantly different between the three groups (Fig. 2c) at the mRNA level. In order to investigate this further, we next compared the expression level and localization of SYCP3 in the untreated and RA treated differentiations. We utilized mouse fetal ovaries as a positive control. Comparing the protein level of SYCP3 in our treated, untreated, and undifferentiated stem cell populations we found the treatment with RA led to significantly higher SYCP3 expression (Fig. 3a). Due to the role of SYCP3 in forming the synaptonemal complex during meiosis it was important to specifically look at how SYCP3 was interacting with the chromosomes. Therefore, following chromosome spreads and immunolocalization of SYCP3 we found the expected phenotype in our fetal ovary controls of synapsed chromosomes labelling positive (Fig. 4b). In our control untreated differentiations positive staining was seen largely as cytoplasmic, suggesting a dysregulation in SYCP3 chromosome loading (Fig. 4b). Interestingly, the treatment with RA resulted in a phenotype more consistent with our fetal ovary control cells (Fig. 4b). Following RA treatment SYCP3 was localized on the chromosomes and not in the cytoplasm, though it is unclear if the chromosomes are fully synapsed. This suggests that RA treatment is improving the transition into meiosis of the OLCs.

During the production of OLCs we noticed that the RA treated OLCs had a thicker zona pellucida-like membrane and were less fragile. Previously we have determined that OLCs only expressed *ZP3* and not *Zp1* and *Zp2* [19]. Therefore, we tested the expression of *Zp1–3* in the differentiations following treatment with RA. We found the expression of factor in the germline alpha *(Figla)* in the untreated and RA treated differentiations as well as in the ovary control (Fig. 5a). FIGLA has been shown to be required for the expression of the ZP genes [32]. While the expression of all three *Zp* transcripts were found following RA treatment and in the ovary control, again *Zp1* and *Zp2* failed to be expressed in our control differentiations (Fig. 5a). We next compared the localization of ZP3 in the untreated and RA treated OLCs (Fig. 5c). ZP3 stained more intensely in the RA treated OLCs and appeared to be more membrane localized and continuous, when compared to the control OLCs (Fig. 5c). This likely explains the less fragile OLCs produced in the RA treated differentiation. It is of note to mention that the majority of the OLCs did not show 100 % continuous ZP3 staining and remained more fragile when being handled when compared to natural oocytes (Fig. 5c). Suggesting the RA treatment does not completely correct ZP membrane issues and potentially prohibiting full functionality in the OLCs.

Finally, we wanted to determine if the treatment of the differentiations resulted in improved OLC production efficiency and growth. While the system remains relatively inefficient, the production of OLCs following RA treatment more than doubled compared to the untreated controls, 18.39 ± 1.58 OLCs per million starting cells compared to 8.17 ± 2,22 OLCs per million starting cells (Fig. 6a). Moreover, the OLCs displayed better growth characteristics following RA treatment, reaching a significantly greater size (Fig. 6a).

## Conclusions

Overall, we have demonstrated that RA can improve the differentiation of skin-derived stem cells into OLCs. It improved the meiotic progress and overall efficiency of the system. Further research will be required to determine if RA treatment improves the functional competency of somatic derived OLCs produced in vitro.

## References

[1] Speed RM (1982). Meiosis in the foetal mouse ovary. I. An analysis at the light microscope level using surface-spreading. Chromosoma.

[2] Bowles J, Knight D, Smith C, Wilhelm D (2006). Retinoid signaling determines germ cell fate in mice. Science.

[3] Baltus AE, Menke DB, Hu Y-CC, Goodheart ML, Carpenter AE, de Rooij DG, Page DC (2006). In germ cells of mouse embryonic ovaries, the decision to enter meiosis precedes premeiotic DNA replication. Nat Genet.

[4] Menke DB, Koubova J, Page DC (2003). Sexual differentiation of germ cells in XX mouse gonads occurs in an anterior-to-posterior wave. Dev Biol.

[5] White JA, Ramshaw H, Taimi M, Stangle W, Zhang A, Everingham S, Creighton S, Tam SP, Jones G, Petkovich M (2000). Identification of the human cytochrome P450, P450RA1-2, which is predominantly expressed in the adult cerebellum and is responsible for all-trans-retinoic acid metabolism. Proc Natl Acad Sci.

[6] Zhou Q, Li Y, Nie R, Friel P, Mitchell D, Evanoff RM, Pouchnik D, Banasik B, McCarrey JR, Small C, Griswold MD (2008). Expression of stimulated by retinoic acid gene 8 (Stra8) and maturation of murine gonocytes and spermatogonia induced by retinoic acid in vitro. Biol Reprod.

[7] Adams IR, McLaren A (2002). Sexually dimorphic development of mouse primordial germ cells: switching from oogenesis to spermatogenesis. Development.

[8] Koubova J, Menke DB, Zhou Q (2006). Retinoic acid regulates sex-specific timing of meiotic initiation in mice. Proc Natl Acad Sci.

[9] Li H, Clagett-Dame M (2009). Vitamin a deficiency blocks the initiation of meiosis of germ cells in the developing rat ovary in vivo. Biol Reprod.

[10] Raverdeau M, Gely-Pernot A, Féret B (2012). Retinoic acid induces Sertoli cell paracrine signals for spermatogonia differentiation but cell autonomously drives spermatocyte meiosis. Proc Natl Acad Sci.

[11] Farini D, Scaldaferri ML, Iona S, Sala LG (2005). Growth factors sustain primordial germ cell survival, proliferation and entering into meiosis in the absence of somatic cells. Dev Biol.

[12] Koshimizu U, Watanabe M, Nakatsuji N (1995). Retinoic acid is a potent growth activator of mouse primordial germ cells in vitro. Dev Biol.

[13] Hübner K, Fuhrmann G, Christenson LK, Kehler J, Reinbold R, De La Fuente R, Wood J, Strauss JF, Boiani M, Schöler HR (2003). Derivation of oocytes from mouse embryonic stem cells. Science.

[14] Geijsen N, Horoschak M, Kim K, Gribnau J, Eggan K, Daley GQ (2004). Derivation of embryonic germ cells and male gametes from embryonic stem cells. Nature.

[15] Toyooka Y, Tsunekawa N, Akasu R, Noce T (2003). Embryonic stem cells can form germ cells in vitro. Proc Natl Acad Sci U S A.

[16] Linher K, Dyce P, Li J (2009). Primordial germ cell-like cells differentiated in vitro from skin-derived stem cells. PLoS One.

[17] Dyce PW, Wen L, Li J (2006). In vitro germline potential of stem cells derived from fetal porcine skin. Nat Cell Biol.

[18] Dyce PW (2013). Differentiation of newborn mouse skin derived stem cells into germ-like cells in vitro. J Vis Exp.

[19] Dyce PW, Liu J, Tayade C, Kidder GM, Betts DH, Li J (2011). In vitro and in vivo germ line potential of stem cells derived from newborn mouse skin. PLoS One.

[20] Dyce PW, Shen W, Huynh E, Shao H, Villagómez DA, Kidder GM, King WA, Li J (2011). Analysis of oocyte-like cells differentiated from porcine fetal skin-derived stem cells. Stem Cells Dev.

[21] Ge W, Ma H-GG, Cheng S-FF, Sun Y-CC, Sun L-LL, Sun X-FF, Li L, Dyce P, Li J, Shi Q-HH, Shen W (2015). Differentiation of early germ cells from human skin-derived stem cells without exogenous gene integration. Sci Rep.

[22] Nayernia K, Nolte J, Michelmann HW, Lee JH (2006). In vitro-differentiated embryonic stem cells give rise to male gametes that can generate offspring mice. Dev Cell.

[23] Zhou Q, Wang M, Yuan Y, Wang X, Fu R, Wan H, Xie M, Liu M, Guo X, Zheng Y, Feng G, Shi Q, Zhao X-YY, Sha J, Zhou Q (2016). Complete meiosis from embryonic stem cell-derived germ cells in vitro. Cell Stem Cell.

[24] Toma J, Akhavan M, Fernandes K, Barnabé-Heider F, Sadikot A, Kaplan D, Miller F (2001). Isolation of multipotent adult stem cells from the dermis of mammalian skin. Nat Cell Biol.

[25] Winterhager E, Kidder GM (2015). Gap junction connexins in female reproductive organs: implications for women's reproductive health. Hum Reprod Update.

[26] Ge W, Cheng SF, Dyce PW, De Felici M, Shen W (2016). Skin-derived stem cells as a source of primordial germ cell-and oocyte-like cells. Cell Death Dis.

[27] Semba RD (2012). On the “discovery” of vitamin a. Annals of nutrition and metabolism.

[28] Rhinn M, Dollé P (2012). Retinoic acid signalling during development. Development.

[29] Suzuki A, Saga Y (2008). Nanos2 suppresses meiosis and promotes male germ cell differentiation. Genes Dev.

[30] Su Q, Sugiura S, Pendola C, Handel S, Eppig (2012). MARF1 regulates essential Oogenic processes in mice. Science.

[31] Su Y-Q, Sun F, Handel M, Schimenti J, Eppig J (2012). Meiosis arrest female 1 (MARF1) has nuage-like function in mammalian oocytes. Proc Natl Acad Sci.

[32] Liang L, Soyal SM, Dean J (1997). FIGalpha, a germ cell specific transcription factor involved in the coordinate expression of the zona pellucida genes. Development.

